# Corneal UV Protective Effects of a Topical Antioxidant Formulation: A Pilot Study on In Vivo Rabbits

**DOI:** 10.3390/ijms21155426

**Published:** 2020-07-30

**Authors:** Marisa Palazzo, Francesco Vizzarri, Lubomir Ondruška, Michele Rinaldi, Luigi Pacente, Germano Guerra, Francesco Merolla, Ciro Caruso, Ciro Costagliola

**Affiliations:** 1 Department of Agricultural, Environmental and Food Sciences, University of Molise, 86100 Campobasso, Italy; m.palazzo@unimol.it; 2 Department of Agricultural and Environmental Sciences, University of Bari Aldo Moro, 70126 Bari, Italy; francesco.vizzarri@uniba.it; 3 National Agricultural and Food Centre, 949 01 Nitra, Slovakia; ondruska@cvzv.sk; 4 Clinica Oculistica, Unicampania, 80131 Napoli, Italy; michrinaldi@libero.it; 5 Corneal Transplant Center, Pellegrini Hospital, 80134 Naples, Italy; pacente@oculum.it; 6 Department of Medicine and Health Science “V. Tiberio”, University of Molise, 86100 Campobasso, Italy; germano.guerra@unimol.it (G.G.); francesco.merolla@unimol.it (F.M.); ciro.costagliola@unimol.it (C.C.)

**Keywords:** UV damage, cornea protection, topic shielding formulation, riboflavin, D-α-tocopherol polyethylene glycol succinate (TPGS), vitamin E

## Abstract

This study aimed to evaluate the protective effect of a topical antioxidant and ultraviolet (UV) shielding action formulation containing riboflavin and D-α-tocopherol polyethylene glycol succinate (TPGS) vitamin E against corneal UV-induced damage in vivo rabbit eyes. In vivo experiments were performed using male albino rabbits, which were divided into four groups. The control group (CG) did not receive any UV irradiation; the first group (IG) was irradiated with a UV-B−UV-A lamp for 30 min; the second (G30) and third (G60) groups received UV irradiation for 30 and 60 min, respectively, and were topically treated with one drop of the antioxidant and shielding formulation every 15 min, starting one hour before irradiation, until the end of UV exposure. The cornea of the IG group showed irregular thickening, detachment of residual fragments of the Descemet membrane, stromal fluid swelling with consequent collagen fiber disorganization and disruption, and inflammation. The cornea of the G30 group showed edema, a mild thickening of the Descemet membrane without fibrillar collagen disruption and focal discoloration, or inflammation. In the G60 group, the cornea showed a more severe thickening, a more abundant fluid accumulation underneath the Descemet membrane with focal detachment, and no signs of severe tissue alterations, as were recorded in the IG group. Our results demonstrate that topical application of eye drops containing riboflavin and TPGS vitamin E counteracts UV corneal injury in exposed rabbits.

## 1. Introduction

Owing to the stratospheric ozone layer depletion, human exposure to ultraviolet (UV) radiation is increasing. Ozone is essential for life on Earth because of its ability to absorb UV radiation. UV can be further divided into four bands: UV-vacuum (100–200 nm); UV-A (400–311 nm); UV-B (310–280 nm); and UV-C (280–100 nm). Eye exposure is wavelength-dependent [1,2]. Approximately 35% of UV-A rays and approximately 80% of UV-B rays have the greatest potential damage [3,4], despite representing less than 1% of the total radiation reaching the Earth’s surface. UV-C radiation is fully absorbed in the upper atmosphere.

Many studies have demonstrated the risk of eye damage induced by broadband solar radiation [5,6]. This is becoming an emerging public health problem, and people should be more aware of the potential risk from ocular exposure to solar UV radiation. Unavoidable exposure to photostress, that is, UV light, visible light, and ionizing radiation, contributes to damage of ocular tissues, mainly triggered by oxidative stress [7].

The ocular surface receives approximately 59–77% of UV light directed to the head. The tear film absorbs wavelengths up to 300 nm, covering much of the UV-B spectrum [8]. In general, shorter wavelengths of UV and visible light penetrate tissues less than longer wavelengths [9,10,11,12]. Sunlight contains much more UV-A than UV-B, and both have no beneficial effects on the eye [13].

The cornea has a high absorption coefficient for UV-B radiation and represents a natural UV-B filter [14], absorbing up to 92% of UV-B at 300 nm, but only 20−40% of UV-A [8,15].

Absorption is age-dependent [16]; in fact, in humans at the age of 8–10 years, UV-A and UV-B radiation easily reaches the retina, with a maximum at 320 nm [17]. The metabolites of tryptophan within the lens absorb UV radiation, decreasing the amount of light transmitted from the lens [18]. By the early 20s, only 0.1% of UV rays can penetrate, and by the age of 60 years, no UV radiation can reach the retina [19], except in the case of aphakia or pseudophakia [20,21].

Ocular tissues and fluids contain enzymatic-catalase, superoxide dismutase, glutathione peroxidase, reductase, nonenzymatic ascorbic acid, reduced glutathione, and alpha-tocopherol antioxidants to protect ocular structures from oxidative damage [22]. However, it has been reported that irradiation of the rabbit eye with UV-A increases corneal hydration and light absorption, while also triggering corneal inflammation [23].

This study aimed to test the protective effect of an antioxidant and shielding action ophthalmic topical formulation against UV damage in corneal tissue of rabbits exposed to UV irradiation.

## 2. Results

**Figure 1:**
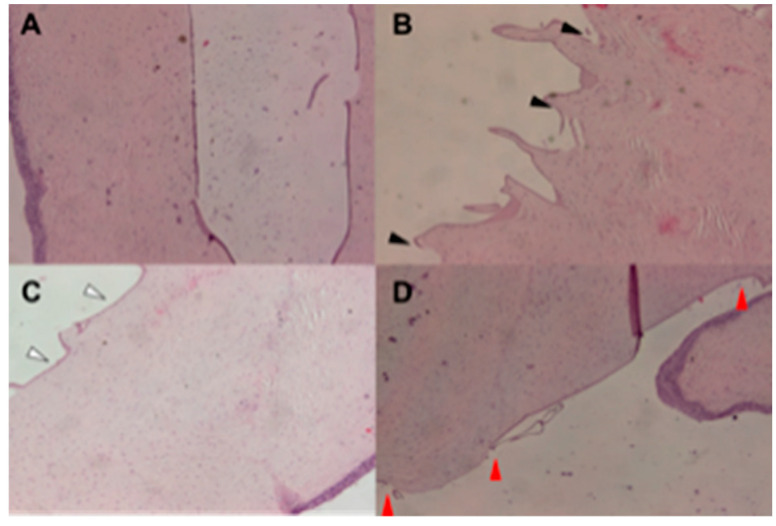
(**A**) Hematoxylin and Eosin (H&E) staining of the corneal tissue obtained from the rabbit eyes of Control Group (CG). Normal corneal thickness and normal morphology of the corneal layers in the control sample. Original magnification × 20. (**B**) H & E staining of the corneal tissue obtained from the rabbit eyes of Irradiated Group (IG). Serious increase in thickness, detachment of residual fragments of Descemet membrane, which appears disappearance for destruction and micropapillary changes (see black arrows). Original magnification × 20. (**C**) H & E staining of the corneal tissue obtained from the rabbit eyes of Irradiated Group (IG30). Moderate increase in thickness, mild edema of the endothelial layer and slight increase in thickness of Descemet membrane (see white arrows). Original magnification × 20. (**D**) H & E staining of the corneal tissue obtained from the rabbit eyes of Irradiated Group (IG60). Severe increase in thickness, detachment of the Descemet membrane and edema of the endothelial layer (see red arrows). Original magnification × 20.

UV irradiation of the cornea caused significant morphologic changes to the exposed rabbits. Histologically, a normal rabbit cornea shows an anterior stratified, squamous, non-keratinizing epithelium sitting on a basement membrane (which resembles the Bowman membrane of humans); under the epithelium there is a large stromal layer (which represents the largest part of the corneal thickness), followed by a membrane that exhibits a homogeneous fine granularity; i.e., Descemet membrane. Lastly, the endothelium is recognized as a flattened layer of cells attached to the Descemet membrane (Figure 1A). Following 30 min UV irradiation (study group IG), we observed irregular corneal thickening, detachment of residual fragments of the Descemet membrane, stromal fluid swelling with consequent collagen fiber disorganization and disruption, and inflammation (Figure 1B). The G30 irradiated rabbit group showed edema, a mild thickening of the Descemet membrane, neither fibrillar collagen disruption and focal discoloration, nor inflammation (Figure 1C). The G60 group showed more severe corneal thickening and greater fluid accumulation underneath the Descemet membrane with focal detachment, as compared to animals from the G30 group (Figure 1D), without the signs of severe tissue alterations found in the irradiated animals not receiving the topical treatment.

## 3. Discussion

Exposure to UV-A irradiation induces apoptosis in rabbit corneal epithelial cells and keratocytes [24]. The degree of damage depends on fluence [22] and wavelength; in fact, wavelengths between 400 and 311 nm caused more extensive damage to the corneal stroma and endothelium than wavelengths between 310 and 280 nm [24]. Thus, UV exposure causes injury to the whole cornea, from the epithelium to the endothelium. Our findings confirm that acute exposure of cornea to UV-A induces cell death in a dose-dependent manner, as reported by previous investigators [25].

Constant exposure to UV induces biological damage in absorbing tissues, mainly consisting of: (i) DNA damage [26], (ii) increase of reactive oxygen species (ROS), and (iii) decrease of antioxidant protective enzymes [27,28]. The peculiar position and function of the eye makes the cornea constantly exposed to UV [29] and the UV light at wavelengths of 270–290 nm or less is totally absorbed by the corneal epithelium and Bowman’s membrane [30].

Lipid peroxidation of cellular membranes, oxidative changes in proteins, direct oxidative damage to DNA [31,32,33], decreasing mitochondrial functions and antioxidant protective mechanisms, lead to apoptosis in corneal tissue [7,34]. The related histologic finding is represented by micropapillary changes and membrane detachment, as occurred in our experiments. Although the exposure of irradiated cornea was about 60 times the maximum exposure of the human cornea to UV-A contained in sunlight [35], the protective treatment was able to counteract the known and expected detrimental effects.

Many known ocular surface and corneal diseases are the direct consequence of UV exposure. For acute events, photokeratitis is the most common, whereas pterygium, pinguecula, and climatic droplet keratopathy are the clinical findings typical of chronic UV exposure [36,37].

Both the corneal epithelium and endothelium, which cannot regenerate, are sensitive to UV radiation. Although reduced glutathione plays a key role in the protection against UV irradiation and highly damaging radicals formed in the process [38], the antioxidant protective mechanisms are vulnerable to increased UV-A exposure; in fact, the reduction of protective species results in corneal damage and injury. In this scenario, we assessed in vivo experiments using a topical application of an ophthalmic preparation containing riboflavin, D-α-tocopherol polyethylene glycol succinate (TPGS) vitamin E, to significantly counteract UV corneal injury in exposed rabbits. The choice of this animal model is based on the statement that the rabbit eye is relatively large; consequently, it has been proven useful for the assessment of both new technologies and ophthalmic surgical procedures in ophthalmic scientific research [39]. Morphologically, the rabbit cornea is very similar to that of humans [40,41]. However, in our recent data [42], the ophthalmic antioxidant and shielding preparation showed a protective role against UV light damage on the retinal tissue of rabbits exposed to UV irradiation. In fact, microscopic analyses of untreated corneas and of corneas treated with UV irradiation resulted in the destruction of all epithelial layers thereby exposing the Bowman membrane, in comparison to the intact cellular architecture of the control group. In corneas treated with UV irradiation without the ophthalmic antioxidant and shielding solution, there were several cellular gaps in the superficial epithelial layer, mostly due to the rupture of intercellular tight junctions. In addition, in corneas treated with UV irradiation, there was a remarkable loss of microvilli and cytoplasmic nuclei. In corneas treated with UV irradiation with the ophthalmic antioxidant and shielding solution, the epithelial layers, cell nuclei, and intercellular tight junctions were less damaged. Moreover, the reduction in microvilli density was less evident and the surviving microvilli appeared morphologically intact. Giblin demonstrated that a soft contact lens with a class I UV block (senofilcon A) prevented the yellow fluorescence induced by UV-A and the loss of NADH in the rabbit lens core in vivo [43].

Vizzari et al. showed that topical application of the antioxidant and shielding solution significantly counteracted the UV-induced oxidative stress in both the aqueous humor and lens of exposed rabbits [40]. Furthermore, riboflavin shows an indirect antioxidant capacity because it has a shielding action (limits the damage caused by UV-A irradiation), as demonstrated in our studies on cross linking [44,45,46].

The existence of ocular biochemical damage due to acute and chronic exposure to UV-B and UV-A has been widely demonstrated. UV-B rays (310–290 nm) and UV-A rays (311–399 nm) are cytotoxic to ocular tissues. This is the main environmental source of photo-oxidation, due to oxidative stress.

Thanks to the cited shielding action, the ophthalmic antioxidant and shielding formulation used in this study is the first and only product certified both as a medical device (Directive 93/42/EEC and subsequent amendments) and personal protective equipment (Directive 89/686/EEC) against UV and blue light. The safety of the corneal cross-linking procedure depends on the addition of more riboflavin during the UV-A irradiation process. The instilled riboflavin forms the tear film on the cornea and absorbs most of the UV-A energy input, thus protecting the ocular tissues. However, the addition of more riboflavin to the corneal surface produces a variable thickness over time of riboflavin on the corneal surface that blocks UV-A absorption in an erratic way and is highly variable over time, acting as a “sun protection” for UV-A transmission during treatment. The thickness of the riboflavin film varies constantly, inducing large variations in UV-A intensity and transmission to the corneal stroma where corneal reinforcement is needed [47].

In the corneal cross-linking (cxl) procedure, the treated cornea is irradiated with UV-A (370 nm) at a variable power between 1 and −3 mW/cm^2^ for 30 min for a total input energy from the UV-A source variable 1.8–5.4. J/cm^2^. These are the values necessary to trigger the oxidation of riboflavin and the formation of free radicals necessary for the creation of interfibrillary bridges. The albedo of a beautiful sunny day that represents 20% [48] of the direct UV intensity on earth is always less than 1 mW/cm^2^. This value does not induce the oxidation of riboflavin, which instead exerts its shield action so much that most of the UV-A absorption comes from unoxidized riboflavin inside the corneal tissue [45,49,50].

In addition, as specified above, TPGS vitamin E is widely used as a drug penetration enhancer through different biological barriers, while exerting a protective effect on biological membranes against free radical damage. Therefore, the effects of TPGS vitamin E on the riboflavin corneal permeability and consequently its protective effect against free radicals have been evaluated.

Since the protective role of the topical antioxidant and shielding action formulation in the anterior segment have been demonstrated, antioxidant and shielding capacity in the cornea was consequently hypothesized. Our studies on the spectrophotometry results of the ophthalmic antioxidant and shielding action formulations are currently under review.

Our data showed a marked increase in corneal epithelium thickness and cell density following exposure to UV radiation (Table 1, Table 2 and Table 3). Both parameters were significantly reduced by topical treatment with ophthalmic antioxidants and shielding preparation, confirming our findings on histological examination. The cumulative UV dose employed in our experiments is hundreds of times higher than those occurring in real life or in exposed outdoor workers [51].

Hwang and Kim [52] recently demonstrated that the transmittance of cross-linked corneas was 10–20% lower than that of control untreated corneas, concluding that riboflavin treatment exerts a protective effect against ultraviolet penetration in the rabbit cornea. However, riboflavin also has antioxidant properties and neutralizes lipid peroxidation throughout the glutathione redox cycle [53]. These two effects could explain the defensive role exerted by riboflavin in our experiments. TPGS vitamin E acts synergistically: TPGS is a synthetic amphiphilic that undergoes enzymatic cleavage to deliver the lipophilic antioxidant TPGS to cell membranes. The antioxidant properties of TPGS are based on cellular enzymatic hydrolysis by cytoplasmic esterases that liberate free α-tocopherol, which then penetrates the cell membrane and through free radical quenching protects the membrane from lipid peroxidation and oxidative damage [54]. Vitamin E TPGS itself, or via the glutathione cycle [55,56,57,58], acts as a potent scavenger of free radicals, that through its lipid-soluble properties is able to play a pivotal role in membrane preservation against lipid peroxidation, blocking the radical chain and creating a low-reactivity derivative unable to attack lipid substrates [59]. UV exposure significantly increases corneal epithelial thickness and cell density. Both parameters were significantly reduced by topical treatment, justifying the morphological picture.

## 4. Materials and Methods

### 4.1. Animals

According to May-Britt Tessem et al. [60], twelve male albino rabbits (New Zealand White, 2.5–3.0 kg) were used. Although the significance of the pharmacological affinity for melanin in intraocular pharmacokinetic studies has been highlighted, most of these studies in the field of ophthalmology have been performed with albino rabbit eyes [61,62]. The trial was performed in the experimental rabbit farm at the National Agricultural and Food Centre, Institute of Small Farm Animal (Nitra, Slovak Republic).

Twelve male albino rabbits (New Zealand White, 2.5–3.0 kg) of the same age were used for in vivo experimental studies. They were divided into four groups of three animals each. Control group (CG) did not receive any irradiation and/or eye drop, while the other three experimental groups were treated as follows: the first group received UV irradiation for 30 min, without receiving eye drop formulation (Irradiation group, IG), the second (G30), and the third (G60) groups received UV radiation for 30 and 60 min, respectively, and were topically treated with one drop (approximately 50 µL) of the antioxidant and shielding formulation every 15 min, starting one h before irradiation, until the end of UV exposure. The study was conducted in accordance with the ARVO Statement for Use of Animals in Ophthalmic and Vision Research, and in accordance with the guidelines of the European Economic Community for Animal Care and Welfare (EEC Law No. 86/609), and was approved in date 28 January 2019 by the Local Ethic Committee (CTS, Department of Medicine and Health Sciences “V. Tiberio”, University of Molise, 15/2019).

At the end of the experiment, the animals were sacrificed by injection of an overdose of sodium pentobarbital, preceded by anesthesia with xylazine (20 mg/kg) and ketamine HCl (5 mg/kg), as previously described [42,43]. Rabbit eyes were quickly enucleated by veterinary doctors provided by the Research Centre. The dissection of each part of the rabbit eye was microscopically performed by an expert ophthalmologist, and the single samples were stored at −20 °C until histochemistry analyses.

### 4.2. UV Irradiation

The animals were euthanized first, 10 min before UV radiation. Rabbits were anaesthetized with an intramuscular injection of xylazine (20 mg/kg) and ketamine HCl (5 mg/kg).

During irradiation, rabbits were confined in a special cage so that only the head remained exposed to UV radiation. The eyes of anaesthetized rabbits were exposed to UV radiation using a Philips medical UV lamp (Serial LTN4006B, Philips S.p.A. 20,126 Milano, Italy) with the following properties: irradiation field 10 cm × 10 cm, one low-pressure lamp at 369 nm (UV-A). The radiant energy was measured with a radiometer (VLX-3W; Cole-Parmer, Vernon Hills, IL, USA). A distance of 7 cm from the cornea was chosen according to the procedure previously described by Giblin et al. [43]. The IG, G30, and G60 groups were exposed to radiation (100 mW/cm^2^) for 30, 30, and 60 min, respectively. The irradiance on the cornea was 100 mW/cm^2^, with a total fluence of 180 and 360 J/cm^2^. Rabbit eyes in the G30 and G60 groups were topically treated with one drop (approximately 50 µL) of the antioxidant and shielding formulation every 15 min, starting one hour before irradiation, until the end of UV exposure.

### 4.3. Ophthalmic Preparation

The ophthalmic antioxidant and shielding preparation consisted of a riboflavin, TPGS vitamin E solution, at pH 7.2, with an osmolarity of 300 mOsm/L (Iromed S.r.l., Italy, patent n^o^ EP 2459186, USP 9192594).

### 4.4. Histochemistry

Cornea samples were fixed in Davidson solution (alcohol 95%, formaldehyde, glacial acetic acid, and distilled water) for 24 h [63].

Rabbit corneal specimens were fixed in buffered 10% formalin, embedded in paraffin, and sectioned. Then, 5 µm thick serial sections of corneal specimens were deparaffinized and treated for hematoxylin and eosin (hematoxylin: Fluka, AG, Switzerland, Buchs SG; Eosin Y: alcohol and water soluble, Winlap, UK) for routine staining, as previously described [64,65,66]. Corneal epithelium thickness was measured with digital image analysis software.

### 4.5. Statistical Analysis

The mean comparison between each pair of studied groups was analyzed using Student’s t-test.

## 5. Conclusions

In conclusion, our findings indicate that an ophthalmic antioxidant and shielding preparation containing riboflavin and TPGS vitamin E is able to counteract the detrimental effects of UV on the rabbit cornea, and its use should be considered as a possible preventive medical treatment.

## Figures and Tables

**Table 1:** Epithelium thickness has been measured with ImageJ software and is expressed in pixels. Values have been normalized on CTRL (unexposed) group and thickness is expressed as fold increase versus CTRL. Descriptive statistic parameters are shown.

	CTRL	IG	G30	G60
Fold increase vs. CTRL	1	1.768667	1.40292	1.676709
Mean	423,600	749,207	594,276	710,254
Median	427,500	759,386	600,771	714,339
MIN	387,000	564,455	536,799	633,306
MAX	456,000	841,418	623,610	751,708
RANGE	69,000	276,963	86,811	118,402

**Table 2:** Student’s *t* test results for each comparison tested.

Student’s *t* Test *p* Value	
0.0000000005	CTRL vs. IG
0.00000000001	CTRL vs. G30
0.000000000000012	CTRL vs. G60
0.00003	IG vs. G30
0.19	IG vs. G60

**Table 3:** Relative cell density as calculated on selected ROI in each sample. Cell count was normalized on cell area and then reported as relative cell density vs. control sample.

	Cell Count.			
	CTRL	IG	G30	G60
Count	33	143	76	141
Area	24,393	49,504	34,611	49,504
Density	0.001352847	0.002889	0.002196	0.002848
Relative Cell Density	1	2.14	1.62	2.11

## References

[1] Cejka C., Luyckx J., Cejková J. (2012). Central corneal thickness considered an index of corneal hydration of the UVB irradiated rabbit cornea as influenced by UVB absorber. Physiol. Res..

[2] Wang F., Gao Q., Hu L., Gao N., Ge T., Yu J., Liu Y. (2012). Risk of eye damage from the wavelength-dependent biologically effective UVB spectrum irradiances. PLoS ONE.

[3] Abengózar-Vela A., Arroyo C., Reinoso R., Enríquez-de-Salamanca A., Corell A., González-García M.J. (2015). In vitro model for predicting the protective effect of ultraviolet-blocking contact lens in human corneal epithelial cells. Curr. Eye Res..

[4] Chaney E.K., Sliney D.H. (2005). Re-evaluation of the ultraviolet hazard action spectrum—The impact of spectral bandwidth. Health Phys..

[5] Chalam K.V., Khetpal V., Rusovici R., Balaiya S. (2011). A review: Role of ultraviolet radiation in age-related macular degeneration. Eye Contact Lens.

[6] Coroneo M. (2011). Ultraviolet radiation and the anterior eye. Eye Contact Lens Sci. Clin. Pract..

[7] Saccà S., Cutolo C., Ferrari D., Corazza P., Traverso C. (2018). The eye, oxidative damage and polyunsaturated fatty acids. Nutrients.

[8] Bashir H., Seykora J., Lee V. (2017). Invisible shield: Review of the corneal epithelium as a barrier to UV radiation, pathogens, and other environmental stimuli. J. Ophthalmic Vis. Res..

[9] Zigman S. (1993). Ocular light damage. Photochem. Photobiol..

[10] Tenkate T.D. (1999). Occupational exposure to ultraviolet radiation: A health risk assessment. Rev. Environ. Health.

[11] Kabuyama Y., Homma M.K., Kurosaki T., Homma Y. (2002). Early signaling events induced by 280-nm UV irradiation. Eur. J. Biochem..

[12] Ma W., Wlaschek M., Tantcheva-Poór I., Schneider L.A., Naderi L., Razi-Wolf Z., Schüller J., Scharffetter-Kochanek K. (2001). Chronological ageing and photoageing of the fibroblasts and the dermal connective tissue. Clin. Exp. Dermatol..

[13] Kochevar I.E. (1989). Cytotoxicity and mutagenicity of excimer laser radiation. Lasers Surg. Med..

[14] Tanaka Y., Nakayama J. (2016). Upregulated epidermal growth factor receptor expression following near-infrared irradiation simulating solar radiation in a three-dimensional reconstructed human corneal epithelial tissue culture model. Clin. Interv. Aging.

[15] Mesa R., Bassnett S. (2013). UV-B-Induced DNA damage and repair in the mouse lens. Invest. Ophthalmol. Vis. Sci..

[16] Behar-Cohen F., Baillet G., de Ayguavives T., Garcia P.O., Krutmann J., Peña-García P., Reme C., Wolffsohn J.S. (2014). Ultraviolet damage to the eye revisited: Eye-sun protection factor (E-SPF^®^), a new ultraviolet protection label for eyewear. Clin. Ophthalmol..

[17] Youssef P.N., Sheibani N., Albert D.M. (2011). Retinal light toxicity. Eye (Lond).

[18] Ortwerth B.J., Bhattacharyya J., Shipova E. (2009). Tryptophan metabolites from young human lenses and the photooxidation of ascorbic acid by UVA light. Invest. Ophthalmol. Vis. Sci..

[19] An M.-J., Kim C.-H., Nam G.-Y., Kim D.-H., Rhee S., Cho S.-J., Kim J.-W. (2018). Transcriptome analysis for UVB-induced phototoxicity in mouse retina. Environ. Toxicol..

[20] Gaillard E.R., Zheng L., Merriam J.C., Dillon J. (2000). Age-related changes in the absorption characteristics of the primate lens. Invest. Ophthalmol. Vis. Sci..

[21] Gaillard E.R., Merriam J., Zheng L., Dillon J. (2011). Transmission of light to the young primate retina: Possible implications for the formation of lipofuscin. Photochem. Photobiol..

[22] Cejková J., Stípek S., Crkovská J., Ardan T., Pláteník J., Cejka C., Midelfart A. (2004). UV Rays, the prooxidant/antioxidant imbalance in the cornea and oxidative eye damage. Physiol. Res..

[23] Cejka C., Pláteník J., Guryca V., Sirc J., Michálek J., Brůnová B., Cejková J. (2007). Light absorption properties of the rabbit cornea repeatedly irradiated with UVB rays. Photochem. Photobiol..

[24] Podskochy A., Gan L., Fagerholm P. (2000). Apoptosis in UV-exposed rabbit corneas. Cornea.

[25] Sidjanin D., Zigman S., Reddan J. (1993). DNA damage and repair in rabbit lens epithelial cells following UVA radiation. Curr. Eye Res..

[26] Hammond B.R., Renzi-Hammond L. (2018). Individual variation in the transmission of UVB radiation in the young adult eye. PLoS ONE.

[27] Cejková J., Stípek S., Crkovská J., Ardan T., Midelfart A. (2001). Reactive oxygen species (ROS)-generating oxidases in the normal rabbit cornea and their involvement in the corneal damage evoked by UVB rays. Histol. Histopathol..

[28] Cejková J., Ardan T., Filipec M., Midelfart A. (2002). Xanthine oxidoreductase and xanthine oxidase in human cornea. Histol. Histopathol..

[29] Buddi R., Lin B., Atilano S.R., Zorapapel N.C., Kenney M.C., Brown D.J. (2002). Evidence of oxidative stress in human corneal diseases. J. Histochem. Cytochem..

[30] Najjar D.M., Awwad S.T., Zein W.M., Haddad W.F. (2006). Assessment of the corneal endothelium in acute ultraviolet keratitis. Med. Sci. Monit..

[31] Vizzarri F., Palazzo M., Bartollino S., Casamassima D., Parolini B., Troiano P., Caruso C., Costagliola C. (2018). Effects of an antioxidant protective topical formulation on eye exposed to ultraviolet-irradiation: A study in rabbit animal model. Physiol. Res..

[32] Cejka C., Cejkova J. (2015). Oxidative stress to the cornea, changes in corneal optical properties, and advances in treatment of corneal oxidative injuries. Oxid. Med. Cell. Longev..

[33] Wakamatsu T.H., Dogru M., Ayako I., Takano Y., Matsumoto Y., Ibrahim O.M.A., Okada N., Satake Y., Fukagawa K., Shimazaki J. (2010). Evaluation of lipid oxidative stress status and inflammation in atopic ocular surface disease. Mol. Vis..

[34] Seen S., Tong L. (2018). Dry eye disease and oxidative stress. Acta Ophthalmol..

[35] Zigman S., McDaniel T., Schultz J.B., Reddan J., Meydani M. (1995). Damage to cultured lens epithelial cells of squirrels and rabbits by UV-A (99.9%) plus UV-B (0.1%) radiation and alpha tocopherol protection. Mol. Cell. Biochem..

[36] Yam J.C.S., Kwok A.K.H. (2014). Ultraviolet light and ocular diseases. Int. Ophthalmol..

[37] Norval M., Cullen A.P., de Gruijl F.R., Longstreth J., Takizawa Y., Lucas R.M., Noonan F.P., van der Leun J.C. (2007). The effects on human health from stratospheric ozone depletion and its interactions with climate change. Photochem. Photobiol. Sci..

[38] Hepel M., Stobiecka M., Peachey J., Miller J. (2012). Intervention of glutathione in pre-mutagenic catechol-mediated DNA damage in the presence of copper(II) ions. Mutat. Res. Fundam. Mol. Mech. Mutagen..

[39] Gwon A. (2008). The rabbit in cataract/IOL surgery. Animal Models in Eye Research.

[40] Delic N.C., Lyons J.G., Di Girolamo N., Halliday G.M. (2017). Damaging effects of ultraviolet radiation on the cornea. Photochem. Photobiol..

[41] Kaye G.I. (1962). Studies on the cornea. III. The fine structure of the frog cornea and the uptake and transport of colloidal particles by the cornea in vivo. J. Cell Biol..

[42] Bartollino S., Palazzo M., Semeraro F., Parolini B., Caruso C., Merolla F., Guerra G., Costagliola C. (2020). Effects of an antioxidant protective topical formulation on retinal tissue of UV-exposed rabbits. Int. Ophthalmol..

[43] Giblin F.J., Lin L.-R., Simpanya M.F., Leverenz V.R., Fick C.E. (2012). A class I UV-blocking (senofilcon A) soft contact lens prevents UVA-induced yellow fluorescence and NADH loss in the rabbit lens nucleus in vivo. Exp. Eye Res..

[44] Rubinfeld R.S., Caruso C., Ostacolo C. (2019). Corneal cross-linking: The science beyond the myths and misconceptions. Cornea.

[45] Caruso C., Epstein R.L., Ostacolo C., Pacente L., Troisi S., Barbaro G. (2017). Customized corneal cross-linking-a mathematical model. Cornea.

[46] Caruso C., Epstein R.L., Troiano P., Ostacolo C., Barbaro G., Pacente L., Bartollino S., Costagliola C. (2019). Topography and pachymetry guided, rapid epi-on corneal cross-linking for keratoconus. Cornea.

[47] Wollensak G., Aurich H., Wirbelauer C., Sel S. (2010). Significance of the riboflavin film in corneal collagen crosslinking. J. Cataract Refract. Surg..

[48] Chapter 3—Meteorological Data. http://www.fao.org/3/X0490E/x0490e07.htm.

[49] Caruso C., Barbaro G., Epstein R.L., Tronino D., Ostacolo C., Sacchi A., Pacente L., Del Prete A., Sala M., Troisi S. (2016). Corneal cross-linking: Evaluating the potential for a lower power, shorter duration treatment. Cornea.

[50] Schumacher S., Mrochen M., Wernli J., Bueeler M., Seiler T. (2012). Optimization model for UV-riboflavin corneal cross-linking. Invest. Ophthalmol. Vis. Sci..

[51] United States Environment Protection Agency Sun Safety. https://www.epa.gov/sunsafety.

[52] Hwang H.S., Kim M.S. (2013). Ultraviolet-visible light spectral transmittance of rabbit corneas after riboflavin/ultraviolet-A (365 nm) corneal collagen cross-linking. Mol. Vis..

[53] Horiuchi S., Hirano H., Ono S. (1984). Reduced and oxidized glutathione concentrations in the lenses of riboflavin-deficient rats. J. Nutr. Sci. Vitaminol..

[54] Constantinides P.P., Han J., Davis S.S. (2006). Advances in the use of tocols as drug delivery vehicles. Pharm. Res..

[55] Costagliola C., Libondi T., Menzione M., Rinaldi E., Auricchio G. (1985). Vitamin E and red blood cell glutathione. Metabolism.

[56] Costagliola C., Iuliano G., Menzione M., Rinaldi E., Vito P., Auricchio G. (1986). Effect of vitamin E on glutathione content in red blood cells, aqueous humor and lens of humans and other species. Exp. Eye Res..

[57] Costagliola C., Menzione M. (1990). Effect of vitamin E on the oxidative state of glutathione in plasma. Clin. Physiol. Biochem..

[58] Caruso C., Porta A., Tosco A., Eletto D., Pacente L., Bartollino S., Costagliola C. (2020). A Novel vitamin E TPGS-based formulation enhances chlorhexidine bioavailability in corneal layers. Pharmaceutics.

[59] Hajibabaei K. (2016). Antioxidant properties of vitamin E. Ann. Res. Antioxidants.

[60] Tessem M.B., Bathen T.F., Čejková J., Midelfart A. (2005). Effect of UV-A and UV-B irradiation on the metabolic profile of aqueous humor in rabbits analyzed by 1H NMR spectroscopy. Investig. Ophthalmol. Vis. Sci..

[61] Fukuda M., Sasaki K. (1990). Changes in the antibacterial activity of melanin-bound drugs. Ophthalmic Res..

[62] Fukuda M., Sasaki K. (1995). Differences between albino and pigmented rabbit eyes in the intraocular pharmacokinetics of sparfloxacin. Drugs.

[63] Toledo C.R., Pereira V.V., Dourado L.F.N., Paiva M.R.B., Silva-Cunha A. (2019). Corosolic acid: Antiangiogenic activity and safety of intravitreal injection in rats eyes. Doc. Ophthalmol..

[64] Nappi C., Di Spiezio Sardo A., Guerra G., Di Carlo C., Bifulco G., Acunzo G., Sammartino A., Galli V. (2004). Comparison of intranasal and transdermal estradiol on nasal mucosa in postmenopausal women. Menopause.

[65] Nappi C., Di Spiezio Sardo A., Guerra G., Bifulco G., Testa D., Di Carlo C. (2003). Functional and morphologic evaluation of the nasal mucosa before and after hormone therapy in postmenopausal women with nasal symptoms. Fertil. Steril..

[66] Rossi A., Pace S., Tedesco F., Pagano E., Guerra G., Troisi F., Werner M., Roviezzo F., Zjawiony J.K., Werz O. (2016). The hallucinogenic diterpene salvinorin A inhibits leukotriene synthesis in experimental models of inflammation. Pharmacol. Res..

